# Strong Increase in Moxifloxacin Resistance Rate among Multidrug-Resistant Mycobacterium tuberculosis Isolates in China, 2007 to 2013

**DOI:** 10.1128/Spectrum.00409-21

**Published:** 2021-12-01

**Authors:** Hui Xia, Yang Zheng, Dongxin Liu, Shengfen Wang, WenCong He, Bing Zhao, Yuanyuan Song, Xichao Ou, Yang Zhou, Susan van den Hof, Frank Cobelens, YanLin Zhao

**Affiliations:** a National Tuberculosis Reference Laboratory, National Center for Tuberculosis Control and Prevention, Chinese Center for Disease Control and Prevention, Beijing, China; b Institute of Hepatology, National Clinical Research Center for Infectious Disease, Shenzhen Third People’s Hospital, Shenzhen, China; c National Institute of Public Health and the Environment, Centre for Infectious Disease Epidemiology and Surveillance, Bilthoven, The Netherlands; d Department of Global Health and Amsterdam Institute for Global Health and Development, Amsterdam University Medical Centers, Amsterdam, The Netherlands; Indian Institute of Science Bangalore

**Keywords:** multidrug-resistant tuberculosis, moxifloxacin, resistance

## Abstract

We designed this study to determine the trend of moxifloxacin resistance among multidrug-resistant tuberculosis (MDR-TB) patients from 2007 to 2013 in China to inform the composition of multidrug-resistant/rifampicin-resistant tuberculosis (MDR/RR-TB) treatment regimens. We assessed moxifloxacin resistance among MDR-TB isolates collected in national drug resistance surveys in 2007 and 2013 that included 3,634 smear-positive and 7,206 culture-positive pulmonary tuberculosis patients, respectively. Moxifloxacin susceptibility was examined by a Mycobacterium growth indicator tube (MGIT) 960 for the 2007 isolates, and by the minimum inhibitory concentration (MIC) method for the 2013 isolates, at both breakpoints 0.5 and 2.0 μg/mL. Risk factors were explored through multivariable log-binominal regression analysis. Mutations in *gyrA* and *gyrB* for part of the isolates were also studied through sequencing. Of 401 MDR strains isolated in 2007, moxifiloxacin resistance could be determined for 319 (79.6%): 41 (12.9%) and 10 (3.1%) were resistant at 0.5 and 2.0 μg/mL, respectively. Of 365 MDR strains isolated in 2013, 338 (92.6%) could be analyzed: 140 (41.4%) and 79 (23.4%) were resistant at 0.5 and 2.0 μg/mL. For patients in 2007, no characteristics were significantly associated with moxifloxacin resistance. For patients in 2013, patients aged ≥60 years (adjusted prevalence ratio [aPR], 1.46; 95% confidence interval [CI], 1.10 to 1.93) were more likely to have resistance at 0.5 μg/mL, whereas those residing in eastern China compared to those in central China had an increased risk of resistance at both 0.5 (aPR, 1.85; 95% CI, 1.38 to 2.48) and 2.0 μg/mL (aPR, 2.14; 95% CI, 1.35 to 3.40). Sequencing results were obtained for 245 and 266 MDR-TB isolates in 2007 and 2013, respectively. In total, 34 of 38 (89.5%) and 89 of 104 (85.6%) of 2007 and 2013 moxifloxacin-resistant (0.5 μg/mL) MDR-TB strains had mutations in the *gyrA* and *gyrB* gene, respectively. Asp94Gly was the most common mutation among 2007 (11 of 38, 28.9%) and 2013 isolates (24 of 104, 23.1%) and conferred high-level moxifloxacin resistance. Moxifloxacin resistance among MDR-TB patients in China increased from modest to high from 2007 to 2013. Moxifloxacin should be used carefully as a potentially effective drug for composing MDR/RR-TB regimens especially for elderly patients in China. Individual susceptibility testing especially rapid molecular-based assays should be conducted to confirm the susceptibility to moxifloxacin.

**IMPORTANCE** China is one of the high-burden countries for multidrug-resistant/rifampicin-resistant tuberculosis (MDR/RR-TB). Moxifloxacin is one of the critical antituberculosis drugs for MDR/RR-TB treatment. Susceptibility to moxifloxacin is therefore very important to compose effective regimens and to provide protection against development of resistance of companion drugs such as bedaquiline and linezolid. There are, however, no nationally representative data on moxifloxacin resistance among MDR/RR-TB cases in China. Therefore, we assessed the resistance prevalence for moxifloxacin among MDR-TB strains isolated in national drug resistance surveys in 2007 and 2013 that covered 72 sites around the country. We demonstrate that the prevalence of moxifloxacin resistance in MDR-TB isolates increased from modest to high, which should prompt the national tuberculosis program to use moxifloxacin cautiously in second-line regimens to treat MDR/RR-TB unless susceptibility can be laboratory-confirmed.

## INTRODUCTION

Globally, there were an estimated 465,000 incident MDR/RR-TB cases in 2019. China is one of the 30 high multidrug-resistant tuberculosis (MDR-TB) burden countries, accounting for 14% of the world’s multidrug-resistant/rifampicin-resistant tuberculosis (MDR/RR-TB) cases (1). The World Health Organization (WHO) guidelines on drug-resistant tuberculosis treatment (2020) recommend that in longer regimens for second-line treatment of MDR/RR-TB patients, all three group A agents and at least one group B agent should be included to ensure that at least four TB drugs are likely to be effective, and at least three drugs are included for the rest of treatment after bedaquiline is stopped. Susceptibility to fluoroquinolones is also one of the eligibility criteria for treating MDR/RR-TB patients with a shorter regimen of 9 to 12 months (2). Moxifloxacin is one of the group A drugs. In most areas of China, drug susceptibility testing (DST) for moxifloxacin is not routinely performed. In view of the absence of routine DST for moxifloxacin for individual patients in most areas in China, data on the background prevalence of moxifloxacin resistance among MDR-TB are important for deciding whether regimens containing moxifloxacin are appropriate in the country. The global proportion of resistance to any fluoroquinolone among MDR/RR-TB cases from the past 15 years in 105 countries was 20.1% (95% confidence interval [CI], 15.5 to 25.0%) (1). Data from the 2007 national TB drug resistance survey (DRS) in China showed that 24.9% of new and 27.5% of previously treated MDR-TB patients were resistant to ofloxacin (3). Resistance to ofloxacin or ciprofloxacin is a poor predictor of resistance to moxifloxacin (4–6). There have been few studies on moxifloxacin resistance among selected MDR-TB cases in China (7–11) and among nationwide 2007 DRS MDR-TB strains with the concentration of 0.25 μg/mL by MGIT (12). Fluoroquinolones have been used widely and accounted for 13.2, 13.4, and 12.6% of all antibiotic prescriptions in 2015, 2016, and 2017, respectively, in China (13–15). Imported moxifloxacin was first clinically introduced and approved in China in 2004. Prescription of moxifloxacin for the treatment of respiratory tract infections increased slowly from 3.5% in 2015 to 4.5% in 2017. Empirical therapy of suspected bacterial infection with fluoroquinolones before tuberculosis diagnosis could delay the diagnosis of active TB and the initiation of treatment (16–20), which may result in ineffective therapy of tuberculosis and subsequent development of fluoroquinolone resistance. The increased use of moxifloxacin in China may thereby bring about increased moxifloxacin resistance of Mycobacterium tuberculosis. The second nationwide DRS was conducted in 2013. Most of fluoroquinolone resistance in M. tuberculosis is attributed to acquisition of mutations within specific regions of *gyrA* and *gyrB*. In this study, we analyzed the prevalence and trend of moxifloxacin resistance, risk factors for resistance, and mutations in *gyrA* and *gyrB* from nationally representative samples of MDR-TB patients in 2007 and 2013 in China.

## RESULTS

### Study population.

A total of 3,634 smear-positive cases and 7,206 culture-positive cases were enrolled in the 2007 survey (3) and the 2013 survey, respectively. Of 7,206 isolates in 2013, 1,874 failed to recover or were contaminated, 317 were identified as nontuberculous mycobacteria. A total of 401 and 365 cases were identified as MDR-TB cases with the proportion method on Löwenstein-Jensen medium in 2007 and with the minimum inhibitory concentration (MIC) method in 2013. Of 401 isolates from 2007, 82 failed to grow in subculture or had no susceptibility results for moxifloxacin after repeating experiments, so 319 strains from 31 provinces were obtained for final analysis. Of 365 isolates from 2013, sociodemographic and clinical data were not available for 27 cases; 338 cases from 29 provinces were finally analyzed.

The mean age (± standard deviation [SD]) of the 319 MDR-TB patients in 2007 was 43.3 (±16.5) years; 206 of 319 (64.6%) were male. The mean age of the 338 MDR-TB cases in 2013 was 46.8 (±16.9) years; 254 of 338 (75.1%) were male. Both the mean age (*P* = 0.008) and the proportion of male MDR-TB cases (*P* = 0.003) increased significantly from 2007 to 2013.

### Moxifloxacin resistance among MDR-TB isolates.

Of 319 MDR-TB strains in 2007, 41 (12.9%) and 10 (3.1%) showed resistance to moxifloxacin by MGIT at 0.5 and 2.0 μg/mL, respectively. Of 338 MDR-TB isolates in 2013, 140 (41.4%) and 79 (23.4%) were shown to be resistant to moxifloxacin by MIC method at 0.5 and 2.0 μg/mL. The proportion of high-level resistance among all moxifloxacin-resistant isolates increased from 10 of 41 (24.4%) in 2007 to 79 of 140 (56.4%) in 2013 (*P* < 0.001).

### MIC distributions of moxifloxacin for 2013 MDR-TB isolates.

**FIG 1:**
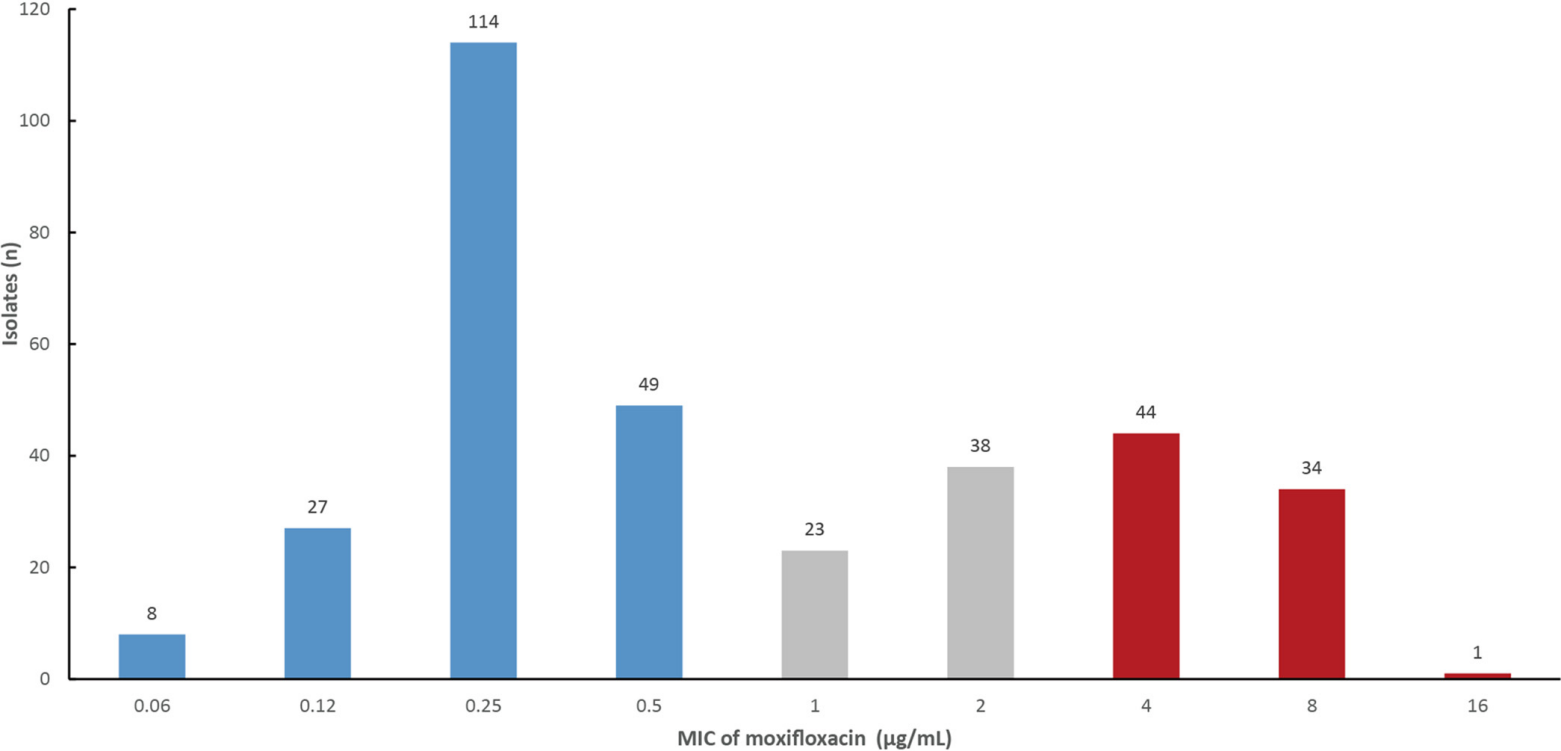
Minimum inhibitory concentration (MIC) of moxifloxacin for 2013 multidrug-resistant isolates. The isolates with growth inhibited at ≤0.5 μg/mL, at 1.0 to 2.0 μg/mL, and at >2.0 μg/mL are marked in blue, gray, and red, respectively.

Of 338 MDR-TB isolates from the 2013 DRS, 198 (58.6%) showed inhibited growth at ≤0.5 μg/mL, 61 (18.0%) showed inhibited growth at 1.0 to 2.0 μg/mL, and 79 (23.4%) showed inhibited growth at >2.0 μg/mL (Fig. 1).

### Cross-resistance between moxifloxacin and ofloxacin.

In 2007, 81 of 319 (25.4%) MDR-TB isolates were resistant to ofloxacin. Of these ofloxacin-resistant isolates, 36 (44.4%) and 9 (11.1%) were also resistant to moxifloxacin at 0.5 and 2.0 μg/mL, respectively. Conversely, 36 of 41 (87.8%) of moxifloxacin-resistant strains at 0.5 μg/mL and 9 of 10 (90%) of isolates resistant at 2.0 μg/mL also showed resistance to ofloxacin.

In 2013, 138 of 338 (40.8%) MDR-TB isolates were resistant to ofloxacin. Of 138 ofloxacin-resistant isolates, 131 (94.9%) and 79 (57.2%) were resistant to moxifloxacin at 0.5 and 2.0 μg/mL, respectively. Conversely, 131 of 140 (93.6%) of moxifloxacin-resistant strains at 0.5 μg/mL and 79 of 79 (100%) of isolates resistant at 2.0 μg/mL were also resistant to ofloxacin (Table 1).

**TABLE 1:** Cross-resistance between moxifloxacin and ofloxacin

Year	Moxifloxacin			Ofloxacin		
	Breakpoint (μg/mL)	Method^*a*^	Susceptibility result	Susceptible	Resistant	Total
2007	0.5	MGIT	Susceptible	233	45	278
			Resistant	5	36	41
			Total	238	81	319
	2.0	MGIT	Susceptible	237	72	309
			Resistant	1	9	10
			Total	238	81	319
2013	0.5	MIC	Susceptible	191	7	198
			Resistant	9	131	140
			Total	200	138	338
	2.0	MIC	Susceptible	200	59	259
			Resistant	0	79	79
			Total	200	138	338

*^a^*MGIT, Mycobacterium growth indicator tube; MIC, minimum inhibitory concentration.

### Factors associated with moxifloxacin resistance.

In multivariable analysis among MDR-TB cases in 2007, farmer occupation and previous usage of fluoroquinolones for antituberculosis treatment showed association with moxifloxacin resistance at 0.5 μg/mL, although both were short of significance (adjusted PR [aPR] for nonfarmers compared to farmers, 0.68 [95% CI, 0.46 to 1.00]; aPR for previous usage of fluoroquinolones, 1.81 [95% CI 0.95 to 3.45]) (Table S1). In multivariable analysis for MDR-TB cases in 2013, patients aged ≥60 years had higher risk for having moxifloxacin resistance at 0.5 μg/mL (aPR for age of ≥60 compared to <40 years: 1.46; 95% CI, 1.10 to 1.93). Patients residing in eastern China were more likely to have moxifloxacin resistance compared with those in central China (aPR for residence in eastern China compared to central China: 1.85 [95% CI, 1.38 to 2.48] and 2.14 [95% CI, 1.35 to 3.40], respectively) (Table 2).

**TABLE 2:** Univariable and multivariable analysis of risk factors for moxifloxacin resistance by MIC at 0.5 and 2.0 μg/mL in 2013^*a*^

Characteristic	All cases, *n*	Resistant at 0.5 μg /mL moxifloxacin						Resistant at 2.0 μg /mL moxifloxacin					
		Resistant, *n* (%)	Susceptible, *n* (%)	cPR		aPR (95% CI)				cPR		aPR (95% CI)	
				Value	*P* value	Value	*P* value	Resistant, *n* (%)	Susceptible, *n* (%)	Value	*P* value	Value	*P* value
Sex													
Male	254	100 (39.4)	154 (60.6)	1				55 (21.7)	199 (78.3)	1			
Female	84	40 (47.6)	44 (52.4)	1.21	0.17	—		24 (28.6)	60 (71.4)	1.32	0.19	—	
Age (yrs)													
<40	125	48 (38.4)	77 (61.6)	1		1		31 (24.8)	94 (61.6)	1			
40 to 59	134	51 (38.1)	83 (61.9)	0.99	0.96	1.00 (0.74 to 1.36)	0.99	29 (21.6)	105 (61.9)	0.87	0.55	—	
≥60	79	41 (51.9)	38 (48.1)	1.35	0.06	1.46 (1.10 to 1.93)	0.009	19 (24.1)	60 (48.1)	0.97	0.90	—	
Region of residence within China													
Central	138	42 (30.4)	96 (69.6)	1		1		21 (15.2)	117 (84.8)	1		1	
East	141	76 (53.9)	65 (46.1)	1.77	<0.001	1.85 (1.38 to 2.48)	<0.001	46 (32.6)	95 (67.4)	2.14	0.001	2.14 (1.35 to 3.40)	0.001
West	59	22 (37.3)	37 (62.7)	1.23	0.34	1.31 (0.88 to 1.96)	0.18	12 (20.3)	47 (79.7)	1.34	0.38	1.34 (0.70 to 2.54)	0.38
Occupation													
Farmer	209	84 (40.2)	169 (59.8)	1				43 (20.6	166 (79.4)	1			
Other	129	56 (43.4)	109 (56.6)	1.08	0.56	—		36 (43.4)	93 (72.1)	1.36	0.12	—	
Number of previous antituberculosis treatments													
0	197	78 (39.6)	119 (60.4)	1				50 (25.4)	147 (74.6)	1			
1	105	46 (43.8)	59 (56.2)	1.11	0.48	—		22 (21.0)	83 (79.0)	0.83	0.40	—	
>1	36	16 (44.4)	20 (55.6)	1.12	0.58	—		7 (19.4)	29 (80.6)	0.77	0.46	—	
Previous fluoroquinolone usage for antituberculosis treatment													
No	331	137 (41.4)	194 (58.6)	1				77 (23.3)	254 (76.7)	1			
Yes	7	3 (42.9)	4 (57.1)	1.04	0.94	—		2 (28.6)	5 (71.4)	1.23	0.73	—	

*^a^*cPR, crude prevalence ratio; aPR, adjust prevalence ratio; CI, confidence interval; “—” , these variables were not included in the final model.

### Mutations of the *gyrA and gyrB* genes in the 2007 and 2013 MDR-TB isolates.

Of 319 2007 DRS isolates, 245 (76.8%) had sequencing results. Of 38 strains resistant at a critical concentration of 0.5 μg/mL, 34 (89.5%) had mutations in the *gyrA* gene, whereas none had mutations in the *gyrB* gene. Of the 207 susceptible strains, 189 (91.3%) showed wild type in *gyrA* and *gyrB*. Mutations in *gyrA* and *gyrB* occurred in sixteen and two susceptible strains, respectively. The most common single mutation in *gyrA* was Asp94Gly (11 of 38, 28.9%). No double mutation was detected. Seven of eight (87.5%) isolates resistant at 2.0 μg/mL had mutations in *gyrA*. Table 3 shows all types of mutations in *gyrA* and *gyrB* gene among the 2007 isolates.

**TABLE 3:** Genotypic mutations in *gyrA* and *gyrB* genes among 2007 multidrug-resistant tuberculosis isolates

Mutation type	Moxifloxacin susceptibility result			
	0.5 μg/mL		2.0 μg/mL	
	Resistant	Susceptible	Resistant	Susceptible
*gyrA*_Ala90Val	7	10	1	16
*gyrA*_Asp94Ala	4	4	0	8
*gyrA*_Asp94Asn	2	0	2	0
*gyrA*_Asp94Cys	1	0	0	1
*gyrA*_Asp94Gly	11	2	2	11
*gyrA*_Asp94His	2	0	1	1
*gyrA*_Asp94Tyr	2	0	0	2
*gyrA*_Ser91Pro	5	0	1	4
*gyrB_*Gly551Arg	0	2	0	2
Wild type	4	189	1	191

Whole-genome sequencing results of 266 (78.7%) strains isolated in 2013 were obtained for analysis. Of 104 isolates resistant at 0.5 μg/mL, 89 (85.6%) had mutations in the *gyrA* or *gyrB* gene. The top three single mutations were Asp94Gly (24 of 104, 23.1%), Ala90Val (19 of 104, 18.3%), and Asp94Tyr (17 of 104, 16.3%). Among 162 susceptible isolates, 157 (96.9%) showed wild type in the *gyrA* and *gyrB* genes. A total of 54 of 58 (93.1%) isolates resistant at 2.0 μg/mL had mutations, whereas 168 (80.8%) of 208 susceptible strains did not show any mutations in the *gyrA* and *gyrB* genes. The most prevalent single mutation among these high-level resistant isolates was Asp94Gly (23 of 58, 39.7%). Of 24 (95.8%) strains with the Asp94Gly mutation, 23 had MICs of ≥4 μg/mL, and 17 of 21 (81.0%) isolates with Ala90Val change had MICs of ≤2.0 μg/mL. The MICs scattered from 1 to 8 μg/mL for Asp94Tyr mutation. Table 4 shows all types of mutations in *gyrA* and *gyrB* genes among the 2013 isolates.

**TABLE 4:** Genotypic mutations in *gyrA* and *gyrB* genes among 2013 multidrug-resistant tuberculosis isolates

Mutation type	Moxifloxacin susceptibility result				Moxifloxacin MIC (μg/mL)								
	0.5 μg/mL		2.0 μg/mL										
	Resistant	Susceptible	Resistant	Susceptible	0.06	0.12	0.25	0.5	1	2	4	8	16
*gyrA*_Ala74Ser	0	1	0	1	0	0	0	1	0	0	0	0	0
*gyrA*_Asp89Asn	1	0	1	0	0	0	0	0	0	0	0	1	0
*gyrA*_Ala90Val	19	2	4	17	0	0	1	1	3	12	3	1	0
*gyrA*_Ser91Pro	1	0	1	0	0	0	0	0	0	0	0	1	0
*gyrA*_Asp94Ala	7	0	1	6	0	0	0	0	1	5	0	1	0
*gyrA*_Asp94Asn	3	0	3	0	0	0	0	0	0	0	0	3	0
*gyrA*_Asp94Gly	24	0	23	1	0	0	0	0	0	1	15	8	0
*gyrA*_Asp94His	2	0	2	0	0	0	0	0	0	0	1	1	0
*gyrA*_Asp94Tyr	17	0	7	10	0	0	0	0	3	7	5	2	0
*gyrA*_Asp461His	1	0	0	1	0	0	0	0	1	0	0	0	0
*gyrB*_Arg446Cys	1	0	0	1	0	0	0	0	1	0	0	0	0
*gyrB*_Asp461Asn	3	1	2	2	0	0	0	1	0	1	2	0	0
*gyrB*_Thr500Asn	1	0	1	0	0	0	0	0	0	0	1	0	0
*gyrA*_Gly88Ala, *gyrA*_Asp94Ala	1	0	1	0	0	0	0	0	0	0	0	1	0
*gyrA*_Gly88Ala, *gyrA*_Asp94Gly	1	0	1	0	0	0	0	0	0	0	0	1	0
*gyrA*_Ala90Val, *gyrA*_Ser91Pro	1	0	1	0	0	0	0	0	0	0	1	0	0
*gyrA*_Ala90Val, *gyrB*_Thr500Asn	1	0	1	0	0	0	0	0	0	0	0	1	0
*gyrA*_Asp94Gly, *gyrA*_Ser91Pro	1	0	1	0	0	0	0	0	0	0	0	1	0
*gyrA*_Asp94Gly, *gyrB*_Asp461Asn	3	0	3	0	0	0	0	0	0	0	3	0	0
*gyrA*_Asp94Ala, *gyrB*_Asn499Thr	1	0	1	0	0	0	0	0	0	0	0	1	0
*gyrA*_Asp94Gly, *gyrB*_Thr500Asn	0	1	0	1	0	0	0	1	0	0	0	0	0
Wild type	15	157	4	168	8	22	94	33	8	3	2	2	0

## DISCUSSION

This is the first study exploring moxifloxacin resistance among MDR-TB cases in China, over time, through two consecutive nationwide representative samples. From 2007 to 2013, a significantly increased moxifloxacin resistance prevalence (at a concentration of 0.5 μg/mL: 12.9% versus 41.4%; at a concentration of 2.0 μg/mL: 3.1% versus 23.4%) was observed among MDR-TB isolates. The proportion of high-level resistance out of all moxifloxacin resistance increased more than 2-fold (24.4% versus 56.4%), indicating that by 2013 over half of moxifloxacin resistance among MDR-TB patients in China was high-level resistance. Moxifloxacin resistance at 0.5 μg/mL and ofloxacin resistance were almost completely concordant. High-level moxifloxacin cross-resistance among ofloxacin-resistant isolates increased from 11 to 57%. Patients aged ≥60 years or residing in eastern China were more likely to have moxifloxacin resistance. More than 85% of strains resistant to moxifloxacin at 0.5 μg/mL isolated in 2007 and 2013 had mutations in the *gyrA* or *gyrB* gene.

Previously reported moxifloxacin resistance prevalence varies substantially by country, ranging from 17 to 62% (21–24). Studies from the Guizhou province and the Xinjiang Uygur Autonomous Region in western China reported prevalence among MDR-TB cases of 21 and 26% (9, 10), respectively. A study from Guangzhou city in eastern China showed 73% moxifloxacin resistance among isolates with resistance to more than one first-line drugs (11). One study also using the 2007 DRS MDR-TB strains reported that moxifloxacin resistance at 0.25 μg/mL by MGIT 960 was 17.4% (12). An increasing trend toward more moxifloxacin resistance among MDR-TB isolates was observed in India, from 60% in 2005 through 2007 up to 80% in 2011 through 2013 (21). The prevalence for moxifloxacin resistance in 2007 in the present study was lower than those reported by other countries, whereas that in 2013 was similar with or higher than most of the reported results. The resistance prevalence in 2007 in the present study was slightly lower than that in the study of Wang et al. (12), which can be explained by their lower breakpoints of 0.25 μg/mL compared with the 0.5- and 2.0-μg/mL breakpoints used in the present study. Imported moxifloxacin was first introduced and approved in China in 2004, and domestic manufactured moxifloxacin products were not approved until 2018. Standardized MDR-TB treatment regimens containing ofloxacin started to be used from 2006 in two pilot cities of the Global Fund to fight AIDS, Tuberculosis and Malaria (GFATM) project in China and then replaced by levofloxacin since 2008 to 2009 because of the higher ofloxacin resistance rate (higher than 20%) among MDR-TB isolates observed in the 2007 DRS (3). In view of the wide usage and probably higher resistance rate for levofloxacin in China, moxifloxacin was added to the MDR-TB regimen as a replacement for levofloxacin and also as a critical drug component in XDR-TB regimens since 2010 in 41 GFATM project pilot cities in 12 provinces. From the national level, the standardized MDR-TB treatment regimens containing levofloxacin or moxifloxacin (as a replacement for levofloxacin) were recommended in the national guidelines from 2012. Thus, more patients are presumed to have the chance of receiving regimens containing moxifloxacin for antituberculosis treatment since then. Therefore, the lower prevalence in 2007 can be explained by the infrequent use of moxifloxacin, especially in peripheral level hospitals, where our study sites were located, because of the high cost and inaccessibility of the drug. We surmise that the increased prevalence 6 years later (2007 to 2013) was attributable to the more frequent usage of moxifloxacin for treatment of common bacterial infections (13–15) or as an antituberculosis drug. The latter was suggested by a study from the national TB referral hospital in China that reported a significant increase in moxifloxacin prescriptions for treating inpatients between 2011 and 2015 (25). The highly increased cross-resistance between ofloxacin and moxifloxacin also indicates wide usage of moxifloxacin during those 6 years.

In 2020, the Chinese national guideline for MDR/RR-TB treatment was updated, with moxifloxacin now being among the critical Group A drugs in standardized MDR/RR-TB regimens. However, given the present resistance prevalence and the availability and increased use of moxifloxacin, we expect that since 2013, its resistance prevalence has further increased. Therefore, the implications for composing of MDR/RR-TB treatment regimens containing moxifloxacin must be considered carefully. Another point to be noted is that more than 50% of moxifloxacin-resistant isolates had MICs of >2 μg/mL in 2013, which indicated that empirically increasing the dosage will not be a sensible option for improving therapeutic effectiveness among moxifloxacin-resistant patients tested at a critical concentration of 0.5 μg/mL. Individual susceptibility testing of moxifloxacin and the level of resistance is thus very necessary given the high background moxifloxacin resistance prevalence in China.

In the present study, no characteristic was associated with moxifloxacin resistance in 2007. In 2013, elderly patients were more likely to have moxifloxacin resistance compared with younger patients, similar with another study in China (11). This may be related to the use of fluoroquinolones, including moxifloxacin, for the treatment of community-acquired pneumonia (CAP). A proportion of patients diagnosed with CAP who are empirically treated with fluoroquinolones will actually have pulmonary TB especially in TB prevalent areas (26–29). Monotherapy with a fluoroquinolone may temporarily improve the patient’s symptoms and, as suggested by previous studies, may thereby delay TB diagnosis and antituberculosis treatment (17–19, 28) and select for fluoroquinolone-resistant M. tuberculosis strains (29–34). In our study, previous usage of fluoroquinolones for antituberculosis treatment was not significantly associated with moxifloxacin resistance, possibly because of the small number of MDR-TB cases with previous usage of fluoroquinolone for treatment of tuberculosis. We hypothesize that elderly patients had more opportunities for exposure to moxifloxacin for treatment of CAP before TB diagnosis. Indeed, two studies also showed that 17 to 35% of TB patients had exposure to fluoroquinolones before diagnosis (31, 35), and elderly patients were more likely than younger patients to be prescribed fluoroquinolones (31). Moxifloxacin resistance in our study was furthermore associated with residence in eastern China. The average economic situation in this region of China is better than that in central and western China, where moxifloxacin may have been less affordable. Unfortunately, we did not collect information on moxifloxacin exposure before TB diagnosis for treatment of other bacterial infections.

The detection of mutations in *gyrA* and *gyrB* genes can help predict the presence and level of moxifloxacin resistance. In the present study, mutations in *gyrA* or *gyrB* were detected in 89.5 and 85.6% of isolates resistant to moxifloxacin at 0.5 μg/mL in 2007 and 2013, respectively. Mutations within the quinolone resistance-determining region (QRDR) of *gyrA* account for 42 to 100% of fluoroquinolone resistance in M. tuberculosis, with codons 90, 91, and 94 being the most mutated sites (36). One study conducted in five countries over the period of 2009 through 2014 showed that the overall pooled sensitivity values for predicting resistance to moxifloxacin by genetic sequencing was 88% (81 to 92%) for *gyrA* and *gyrB* (24). Different mutations in *gyrA* are associated with different levels of moxifloxacin resistance. Kambly et al. reported that strains with mutations of Ala90Val or Ser91Pro had an MIC of 1.0 μg/mL for moxifloxacin, whereas isolates with mutations at Asp94Ala, Asp94Asn/Tyr, Asp94Gly, and Asp94His were associated with an MIC of 2.5 μg/mL (37). In the present study, *gyrA* mutations at codon 94, particular Asp94Gly, were the most common mutations conferring moxifloxacin resistance. The majority of the 2013 strains (95.8%) with the Asp94Gly mutation conferred high-level resistance, whereas most isolates (81.0%) with a Ala90Val change conferred low-level resistance. Asp94Tyr cannot distinguish the low- and high-level moxifloxacin resistance clearly. Of 17 2007 strains with Ala90Val mutation, 10 (58.8%) showed phenotypically susceptible result at a critical concentration of 0.5 μg/mL. Several other studies also showed that most of the clinical isolates with the Ala90Val mutation had MICs of 0.5, 0.25, or 1 μg/mL, at or close to the critical concentration of 0.5 μg/mL, in the MGIT 960 system (37–40). We infer that these strains may have had borderline MICs, which were detected and interpreted as susceptible with the critical concentration of 0.5 μg/mL. Decreasing the critical concentration from 0.5 to 0.25 μg/mL in the MGIT 960 system as described in the 2018 WHO technical report (41) could resolve this category of borderline resistance to some extent. However, a lower critical concentration will also probably result in lower sensitivity of *gyrA*- and *gyrB*-based mutations to detect moxifloxacin resistance, as evidenced by the finding that only 54.4% of moxifloxacin-resistant strains at an MIC of 0.25 μg/mL in the 2007 DRS MDR-TB isolates had mutations in *gyrA* and *gyrB* (12), compared with 89.5% at an MIC of 0.5 μg/mL in the present analysis. This warrants more studies combining genotypic and phenotypic methods.

The strength of the present study is that it included MDR-TB isolates from around almost the entirety of mainland China (31 of 31 provinces in 2007 and 29 of 31 provinces in 2013). The trend of resistance of moxifloxacin during a period of several years was explored. There were also some limitations. The 2013 survey used the same clusters as the 2007 survey; whereas the 2007 survey population was a representative survey for all of the 31 provinces included, the 2013 survey may not completely represent the whole country. Not all MDR-TB strains isolated from the survey could be included for the final analysis, which possibly introduced selection bias. The sample size of the original study was not set to study geographic, clinical, and demographic variation for moxifloxacin resistance, so our study may have failed to detect true differences in this regard. We did not adjust the standard errors for the cluster design, thereby potentially overestimating the statistical significance of associations. Two different phenotypic DST methods were used for testing the resistance to moxifloxacin among 2007 and 2013 MDR-TB isolates because the experiments were conducted at different times. The critical concentration and clinical breakpoint for moxifloxacin susceptibility testing used for 2007 isolates were one dilution higher than those updated by the WHO at the end of 2018 (42). Therefore, the moxifloxacin resistance prevalence in 2007 based on the currently used concentration (0.5 μg/mL) was slightly lower than that tested on the lower concentration (0.25 μg/mL) as reported previously (12). A commercial plate-based MIC method was used for the 2013 isolates. There is no definite breakpoint for moxifloxacin recommended by the WHO or the Clinical Laboratory and Standards Institute (CLSI). Finally, the strains and data were isolated and collected years ago, so the results may not accurately reflect the current levels of resistance despite of the observed increased trend. Hence, analysis of more recently collected strains is important to confirm the effect of moxifloxacin resistance for MDR-TB patients currently treated in China especially against the background of limited availability of individual susceptibility testing of moxifloxacin resistance.

In conclusion, the prevalence and level of moxifloxacin resistance in MDR-TB isolates increased from modest to high in China in only 6 years. Mutations in *gyrA* and *gyrB* had higher sensitivity of detection of moxifloxacin resistance. Moxifloxacin therefore should be used with caution as a potentially effective drug for composing MDR/RR-TB regimens especially for elderly patients in China unless susceptibility can be confirmed by molecular or phenotypic methods. Individual moxifloxacin susceptibility testing, especially rapid molecular-based assays, should be recommended in the national tuberculosis program in China.

## MATERIALS AND METHODS

### Study population.

The M. tuberculosis isolates and data used in this study were collected as part of two national DRSs: one among notified smear-positive TB patients in 2007 that has been described elsewhere (3) and the other among notified culture-positive TB patients in 2013. Both surveys followed a cluster design; the 2013 DRS included the same 70 counties or districts as clusters as the 2007 survey. Newly registered culture-positive TB patients with written informed consent were enrolled from January to December in 2013. Culture-positive isolates were transferred to the national tuberculosis reference laboratory to conduct M. tuberculosis strain identification by *p*-nitrobenzoic acid (PNB) medium and DST using the 7H9 MIC method.

### Patient information.

Patient data were collected from the questionnaires used in the 2007 and 2013 surveys, including age, sex, occupation, area of residence within China, previous anti-TB treatment episodes, and previous usage of fluoroquinolones for antituberculosis treatment. The areas of residence were classified into three categories (east, central, and west) based on the geographical positions and economic levels.

### Phenotypic drug susceptibility testing.

Susceptibility to moxifloxacin for the 2007 isolates was determined with the Bactec Mycobacterium growth indicator tube (MGIT) 960 system (Becton Dickinson) at a critical concentration of 0.5 μg/mL and a clinical breakpoint of 2.0 μg/mL as recommended by the WHO in 2012. Susceptibility testing to ofloxacin had been performed earlier using the proportion method on Löwenstein-Jensen (L-J) medium at a critical drug concentration of 4.0 μg/mL (3). For the 2013 isolates, susceptibility was tested by the commercial Sensititre MycoTB plate with concentrations ranging from 0.12 to 16 μg/mL for rifampicin, 0.03 to 4 μg/mL for isoniazid, 0.06 to 16 μg/mL for moxifloxacin, and 0.25–32 μg/mL for ofloxacin. M. tuberculosis growth in the wells with drugs was evaluated visually using the Vizion instrument in comparison with the growth in a drug-free control well on days 14 through 21. Categories of resistance were made based on the proposed breakpoints by the CLSI at ≥2 μg/mL for rifampin and ≥0.25 μg/mL for isoniazid (43). The definitions of resistance for ofloxacin and moxifloxacin were higher than 2.0 μg/mL for ofloxacin and 0.5 and 2.0 μg/mL for moxifloxacin, respectively, as described in previous studies (44–46). Moxifloxacin resistance was classified as high-level resistance according to the breakpoint of 2.0 μg/mL. M. tuberculosis H37Rv was included in each batch of DST. When the result of M. tuberculosis H37Rv was reported as susceptible by MGIT 960 or MIC ranges ≤0.5 μg/mL by a Sensititre MycoTB plate, the results in the same batch were considered valid (43). Otherwise, the experiments were repeated.

### Sequencing of *gyrA* and *gyrB* genes.

Sequencing results for parts of the strains were obtained and included for retrospective analysis. MDR isolates from 2007 were sequenced for the quinolone resistance-determining regions (QRDR) of the *gyrA* and *gyrB* genes. The following forward and reverse primer pairs were used as described previously (47): *gyrA*-F, CAG CTA CAT CGA CTA TGC G; *gyrA*-R, GGG CTT CGG TGT ACC TCA T; *gyrB*-F, GTC GTT GTG AAC AAG GCT GTG; and *gyrB*-R, GTG GAA ATA TGT TGG CCG TC. Sequences were aligned with the homologous sequences of the reference M. tuberculosis H37Rv strain. The expected size of the PCR products was 320 bp (+78 to +397) for *gyrA* and 412 bp (+1330 to +1742) for *gyrB*. PCR was performed in a total volume of 25 μL. The PCRs were performed under the following conditions: initial denaturation at 94°C for 5 min followed by 40 cycles of denaturation at 94°C for 1 min, annealing at 60°C for 1 min, and extension at 72°C for 1 min. For MDR isolates from 2013, whole-genome sequencing was used. The whole genomic DNA was extracted using cetyltrimethylammonium bromide (CTAB) method for next-generation whole-genome sequencing based on 2 × 150 paired-end configuration by Illumina HiSeq 2500 system (Illumina Inc., San Diego, CA). The mutations of *gyrA* and *gyrB* were analyzed in comparison with M. tuberculosis H37Rv (GenBank accession number NC_000962.3) with the online tool TB Profiler (https://tbdr.lshtm.ac.uk/) in January 2021.

### Statistical analysis.

Continuous variables were presented as the mean with standard deviation (SD). Means of two continuous variables were compared by Student’s *t* test with independent samples. Categorical variables were summarized by presenting the frequency and proportion. The frequencies of categorical variables were compared using Pearson or Fisher’s exact test when appropriate. Moxifloxacin-resistant cases at 0.5 μg/mL were compared with those moxifloxacin-susceptible cases with respect to patient characteristics separately for each survey, using univariable and multivariable log-binominal regression to calculate unadjusted and adjusted prevalence ratios (PR) for factors associated with moxifloxacin resistance. Age and gender were fixed, and other characteristics with a *P* value of <0.1 were included in the initial multivariable regression model. The final model was determined using backward selection guided by statistical significance (*P* value of <0.05). The data were analyzed using IBM SPSS software, version 22.0.

### Ethical approval.

The 2007 and 2013 DRS were ethically approved by the Ethical Committees of the Chinese Center for Disease Control and Prevention. Ethics approval of the present study was waived because all isolates were from previous DRS, patient information was extracted from the previous questionnaires, and no additional data and specimens were collected.
