# Supplementary material for: Strong Increase in Moxifloxacin Resistance Rate among Multidrug-Resistant Mycobacterium tuberculosis Isolates in China, 2007 to 2013
Source: Microbiol Spectr. 2021 Dec 1;9(3):e00409-21. doi: 10.1128/Spectrum.00409-21 (PMC8635133; doi:10.1128/Spectrum.00409-21)
Supplement: SUPPLEMENTAL FILE 2 — Supplemental material. Download SPECTRUM00409-21_Supp_2_seq7.pdf, PDF file, 0.1 MB [file spectrum00409-21_supp_2_seq7.pdf]

Strong increase in moxifloxacin resistance rate among multi-drug resistant *Mycobacterium tuberculosis* isolates in China, 2007-2013

Table S1. Univariable and multivariable analysis of risk factors for moxifloxacin resistance at 0.5µg/mL by MGIT in 2007

Characteristic	All cases n	Resistant n (%)	Susceptible n (%)	cPR (95% CI)	*P value*	aPR (95% CI)	*P value*
Gender							
Male	206	25 (12.1)	181 (87.9)	1			
Female	113	16 (14.2)	97 (85.8)	1.10(0.77-1.58)	0.61	-	
Age (years)							
＜40	150	14 (9.3)	136 (90.7)	1			
40-59	106	16 (15.1)	90 (84.9)	1.33(0.89-2.0)	0.16	-	
≧60	63	11 (17.5)	52 (82.5)	1.47 (0.93-2.32)	0.10	-	
Residential area within China							
East	103	12 (11.7)	91 (88.3)	1			
Central	159	20 (12.6)	139 (87.4)	1.05 (0.70-1.57)	0.82	-	
West	57	9 (15.8)	48 (84.2)	1.21 (0.73-2.0)	0.46	-	
Occupation							
Farmer	200	31 (15.5)	169 (84.5)	1		1	
other	119	10 (8.4)	109 (91.6)	0.70 (0.47-1.02)	0.07	0.68 (0.46-1.00)	0.05
Number of previous anti-tuberculosis treatments							
0	130	16 (12.3)	114 (87.7)	1			
1	105	11 (10.5)	94 (89.5)	0.91 (0.59-1.39)	0.66	-	
> 1	80	13 (16.2)	67 (83.8)	1.19 (0.78-1.83)	0.42	-	
unknown	4	1 (25.0)	3 (75.0)	1.63 (0.42-6.35)	0.49	-	
Previous fluoroquinolones usage for anti-tuberculosis treatment							
No	300	36 (12.0)	264 (88.0)	1		1	
Yes	19	5 (26.3)	14 (73.7)	1.72 (0.91-3.24)	0.09	1.81 (0.95-3.45)	0.07

cPR: crude Prevalence Ratio; aPR: adjust Prevalence Ratio
